# Prosumer capitalism in the sharing economy: a gender approach to service providers’ experiences in ridesharing platforms

**DOI:** 10.3389/fsoc.2023.1274969

**Published:** 2024-01-05

**Authors:** Fernando Rey Castillo-Villar, Rosalia G. Castillo-Villar, Krystel K. Castillo-Villar

**Affiliations:** ^1^Facultad de Ciencias Económicas y Empresariales, Universidad Panamericana, Mexico City, Mexico; ^2^Universidad Anáhuac Mayab, Mérida, Mexico; ^3^Business School, Universidad Anáhuac Mayab, Mérida, Mexico; ^4^Deparment of Mechanical Engineering and Texas Sustainable Research Institute, University of Texas at San Antonio, San Antonio, TX, United States

**Keywords:** prosumer behavior, prosumer capitalism, sharing economy, gender, service providers

## Abstract

**Introduction:**

The study draws on the theory of “prosumer capitalism” to explore the experiences of female drivers in ridesharing platforms.

**Methods:**

Twenty-five phenomenological in-depth interviews were carried out with Mexican female drivers in ridesharing platforms.

**Results:**

The results yielded insights regarding the motives of women to become rideshare drivers, their prosumption experiences, and gender issues related to the job.

**Discussion:**

The study offers a novel gender-based approach to comprehend the status of female service providers as prosumer-as-producers and the diverse risks and challenges they face while working in the sharing economy. In a practical sense, platform designers and marketers can improve the application functions to attend to the specific needs of female drivers and implement inclusive measures to safeguard their integrity and well-being.

## 1 Introduction

The sharing economy (SE), defined as “a scalable socio-economic system that employs technology-enabled platforms to provide users with temporary access to tangible and intangible resources that may be crowdsourced” (Eckhardt et al., 2019, p. 3), has attracted an increasing amount of attention from scholars, practitioners, media and policy makers due to its disruptive impact across diverse industry sectors such as hospitality, transportation and retail (Buhalis et al., 2020). The SE has also altered the traditional marketing views of consumers, firms and regulatory entities (Eckhardt et al., 2019; Luo et al., 2021). Recent SE literature has emphasized and endorsed the economic, social and environmental benefits provided by this new economy (Klarin and Suseno, 2021). However, the negative consequences of the SE have been discussed far less frequently (Köbis et al., 2021).

Different authors have raised concerns about the “dark side” of the SE and the urgent need to address issues related to regulatory policy, working conditions and data privacy (Acquier et al., 2017; Murillo et al., 2017; Belk et al., 2019; Etter et al., 2019). In particular, the negative implications of the labor status of service providers in the SE has become an important topic in recent years (Klarin and Suseno, 2021). Within a broader call for research on the paradoxes of the SE, Eckhardt et al. (2019) noted that the precarious condition of the SE workforce is a phenomenon that deserves more in-depth understanding. Following this research stream, the main aim of this study is to explore and comprehend the experiences of female drivers on ridesharing platforms through the theoretical lens of prosumer capitalism.

First, ride-sourcing has grown exponentially worldwide, and multiple ridesharing platforms have been created (e.g., Uber, Lyft and Didi) to meet this demand (Jin et al., 2018). Notwithstanding, ridesharing platforms have also been criticized for their exploitative treatment to service providers, and even the term “Uberization” has been coined to refer to the precarity of labor in other industries (see Davis and Sinha, 2021). Second, the theory of prosumer capitalism (Ritzer, 2015) enables a better understanding of the position of service providers in the SE as they simultaneously consume and produce to deliver the service. The term “prosumer” was also adopted by Bardhi and Eckhardt (2017) to explain the fluidity of the individual to move freely between the role of consumer and producer in the SE, and the same authors have invited future research on “the subject position of the prosumer-entrepreneur” (p. 591). Third, there is a paucity of research regarding gender in the SE, despite its relevance in the area (Hossain, 2020). In particular, scant attention has been given to questions of gender and identity in SEs (Schoenbaum, 2016).

This paper is structured as follows. In the next section, the theoretical foundations upon which this research is based are presented. This section encompasses the literature on the paradox of the SE, prosumer capitalism, and the role of gender in the SE. The methodology introduces a thorough explanation of the use and application of in-depth phenomenological interviews to women drivers in ridesharing services. In the analysis of the results, the data gathered from the interviewees are laid out through a model divided into 3 major themes. Finally, discussions and conclusions address the theoretical and practical implications of the study, as well as potential lines for future research.

## 2 Literature review

### 2.1 The paradox of the sharing economy

Over the last decade, the term “sharing economy” (SE) has become widely popular in the media and public. Indeed, SE could set a new stage in the evolution of economic progress (Trenz et al., 2018). The disruptive effect and dominance of Uber and Airbnb in the long-established transportation and hospitality sectors are proof of the SE’s worldwide popularity and economic success (Zervas et al., 2017; Reich and Yuan, 2019). It is expected that in 2025, the revenues of SE companies will reach an estimated 335 billion dollars (PwC, 2015). Scholars have also shown considerable interest in the SE, and studies have proliferated from different disciplines, including anthropology, economics, marketing, geography, law, innovation, psychology, sociology, sustainability, tourism and management (Hossain, 2020).

However, despite the exponential growth of SE studies in the last few years (Hossain, 2020), there is still a lack of agreement among scholars regarding its conceptualization (Acquier et al., 2017; Netter et al., 2019). First, SE has been labeled with different terms, such as collaborative consumption (Botsman and Rogers, 2010), access-based consumption (Bardhi and Eckhardt, 2012), commercial sharing (Lamberton and Rose, 2012), mesh (Gansky, 2010) and platform economy (Kenney and Zysman, 2016). For the purpose of clarity, SE will be the term adopted in the present article, as it is the most commonly used term in the literature (Ranjbari et al., 2018). Additionally, drawing from the work of Eckhardt et al. (2019), SE will be understood as “a scalable socioeconomic system that employs technology-enabled platforms to provide users with temporary access to tangible and intangible resources that may be crowdsourced” (p. 3).

Second, and the primary concern of this article, numerous debates have arisen regarding the ambiguous use and interpretation of the term “sharing” in the extant SE literature. According to Belk (2007), sharing is “the act and process of distributing what is ours to others for their use and/or the act and process of receiving or taking something from others for our use” (p. 126). As an alternative to private ownership, sharing is a prosocial behavior that provides temporary access to goods and services. Thus, sharing is different from gift-giving and market exchanges, in which there is a transfer of ownership from one person to another (Belk, 2010). However, sharing also differs from renting in that the former does not require a monetary exchange to be performed, thereby operating outside the logic of the market (Belk, 2014; Eckhardt and Bardhi, 2016).

As a nonmarket-mediated practice, sharing is a form of social exchange sustained by existing relationships and trust among the members of a social group (Eckhardt and Bardhi, 2016) through which social collaboration and community building are fostered (Habibi et al., 2017). However, most of the current SE practices are profit-oriented and guided by the market norms of reciprocity (Akbar, 2019). Therefore, SE has been considered “pseudo sharing” rather than true sharing (Belk, 2014; Acquier et al., 2017; Habibi et al., 2017). Habibi et al. (2017) argue that some SE practices may fall into the category of “pseudo sharing,” as they deprioritize community building and emphasize economic benefits. In a similar vein, Küper and Edinger-Schons (2020) claim that SE platforms, such as Airbnb and Uber, are distant from the social nature of sharing since these are short-term rental activities that “belong to the realm of market exchanges” (p. 223).

This “pseudo sharing” dilemma is present in the “feel-good” story widely adopted by SE companies, in which values associated with trust, sharing, community and equity are emphasized, and SE is championed as a sustainable alternative to market capitalism (Acquier et al., 2017; Frenken and Schor, 2019; Gerwe and Silva, 2020). Nevertheless, the profit orientation of the most important SE platforms hinders social cohesion, community building and prosocial behavior (Ritter and Schanz, 2019). Hence, some authors claim that SE replicates and intensifies the inequalities generated by the capitalist system (Acquier et al., 2017; Murillo et al., 2017; Etter et al., 2019). Belk et al. (2019) gave the name “the paradox of the SE” to the dual and contrasting logic derived from the marketization of sharing. Saravade et al. (2020) follow the same logic by arguing that the SE is an extension of the capitalist market system, rather than a scape from it.

The paradoxical nature of the SE has opened discussions on its deficiencies and negative impacts on the economy, society, and environment. Murillo et al. (2017) set a critical research agenda on the controversies of the SE regarding the market (disruption to traditional business models), the government (lack of taxation and regulation to SE platforms), workers (precarious working conditions), consumers (questionable privacy mechanisms) and environment (ecological footprint greater than conventional shopping). Etter et al. (2019) also stressed moral and ethical concerns related to privacy, worker rights, governance and regulation. As Eckhardt et al. (2019) noted, it is crucial to expand our knowledge about the dark side of SE. Therefore, the current study responds to this call by exploring the paradox of SE through the perspectives of female prosumers. However, first it is necessary to conceptualize the prosumer and comprehend its position in the paradox of the SE.

### 2.2 The paradoxical nature of the prosumer in the SE

The term prosumer was coined by Alvin Toffler in 1980. In his book, *The Third Wave*, Toffler (1980) refers to prosumers as people who produce what they consume. Even though prosumption was common in preindustrial societies, the Industrial Revolution separated the production and consumption processes. In an optimistic view, Toffler (1980) predicted the return of prosumption in contemporary society due to advancements in information technology, democratization, and international trade. The discussion on prosumption was forgotten for over two decades, but the emergence of the Internet, especially Web 2.0, has sparked renewed interest by scholars on the topic (Hamalainen et al., 2018). Since then, subsequent studies have expanded the conceptualization of prosumption (Ritzer and Jurgenson, 2010; Cova and Cova, 2012; Ritzer et al., 2012; Rifkin, 2014; Ritzer, 2015).

This article draws on the “prosumption continuum” framework proposed by Ritzer (2015). In contrast with Toffler (1980), Ritzer (2015) contends that there is no such thing as pure production or pure consumption in societies, but that both functions are present all the time in the form of prosumption. Hence, “there can never be any production without consumption” (Ritzer, 2015, p. 416). The prosumption continuum supports this idea by presenting two ends: prosumption-as-production (p-a-p) and prosumption-as-consumption (p-a-c). According to Ritzer (2015), p-a-p involves producers, that is, workers who consume raw material to produce goods. In turn, p-a-c involves consumers who also perform production functions to acquire products/services (e.g., self-serving in fast-food restaurants and ATMs). It is noteworthy that for Ritzer (2015), producers (workers) and consumers are both prosumers who produce and consume at relatively the same time.

Ritzer (2015) used the “prosumption continuum” to explain the evolution of capitalism and its exploitative dynamic through three stages (Table 1). The SE unfolds within the context of the last stage, prosumer capitalism, in which prosumers (workers and consumers) are more susceptible to being exploited and alienated by the doubly exploitative capitalist system (Ritzer, 2015). This type of exploitation is possible because most SE offerings are simultaneously co-created by producers and consumers through digital platforms owned by firms. Indeed, it can be argued that the division between consumption and labor in the SE is blurred (Lan et al., 2017). Ritzer’s (2015) critique of the expansion of prosumer capitalism to the SE is similar to that of the paradox of the SE. Although workers and consumers are both potentially affected by the dark side of the SE, this article is focused specifically on service providers (prosumption-as-producers) and the challenges they face in the SE.

**Table 1:** The evolution of prosumer capitalism.

Type of capitalism	Exploitation system	Producers (p-a-p)	Consumers (p-a-c)	Position in the prosumption continuum	Space–time of the exploitation
Producer capitalism (19th century)	Singly exploitative producer capitalism	Economic success of capitalism in exploiting the proletariat. Low wages, poor work conditions and lack of labour rights.	Minimum level of exploitation.	Dominated by p-a-p	P-a-p in factories and p-a-c in markets.
Consumer capitalism (20th century)	Doubly exploitative consumer capitalism	Exploitation persists with low wages and extended periods of work.	Consumers are exploited through hyper-consumption induced by the financial and marketing systems. Consumers buy more than they need and have more access to credit cards.	Dominated by p-a-c	P-a-p in factories and offices and p-a-c in shopping venues.
Prosumer capitalism (21st century)	Synergistically doubly exploitative prosumer capitalism	Exploitation persists along with technological advances that have made workforce more dispensable.	Consumers are put to work by firms without receiving a economic reward for it (e.g., self-service systems).	In the middle point of the continuum	Exploitation takes place often at the same setting and time.

From an optimistic view, SE has been considered a way through which service providers can become empowered entrepreneurs enjoying flexible schedules and freedom (Etter et al., 2019). However, working conditions in the SE are far from being beneficial to prosumers on the production side. First, service providers are not well compensated by SE firms. In comparison with full-time employees in traditional companies, service providers do not receive benefits (e.g., health insurance or retirement), incur additional expenses (e.g., Uber drivers paying for gasoline and Airbnb hosts covering damage costs) and are susceptible to a high level of income instability, as SE firms can make drastic changes to the pricing mechanism (Jin et al., 2018). All of this has caused multiple public expressions of discontent by service providers about the marginal wages they receive from their labor (Eckhardt et al., 2019).

Second, the relationship between service providers and SE firms presents “an uneven distribution of costs and liabilities” (Murillo et al., 2017). Service providers’ status as independent contractors entails that SE firms are not compelled to offer them benefits or labor protections (Hagiu and Wright, 2019). Hence, SE firms find it convenient to categorize service providers in this way since it “…saves money and removes much of the legal liability from issues arising out of work” (Murillo et al., 2017). Moreover, even though this employment classification offers service providers the flexibility to decide where and when to work, it also involves that they must invest their own capital (Healy et al., 2017), which can disrupt their consumption orientations and their relationships with their possessions (Bardhi and Eckhardt, 2017).

Last, the exploitative nature of SEs to service providers tends to worsen economic and social inequalities (Chai and Scully, 2019). A similar remark was exposed by Ganapati and Reddick (2018), who affirmed that the SE can be considered “a harsher form of capitalism that could exacerbate inequality” (p. 85). As mentioned above, the lack of labor benefits and the highly competitive SE market leave service providers with marginal wages. This situation generates income inequality and goes in the opposite direction of the SE’s promise of equality and societal welfare (Eckhardt et al., 2019). The SE also tends to replicate inequalities based on race, class, and gender (Schor, 2016; Acquier et al., 2017; Schor and Attwood-Charles, 2017). Despite the relevance of all these types of inequalities, the current study will be particularly centered on gender inequality to service providers in the SE context.

### 2.3 A gender equality approach to p-a-p in the SE

In 2015, the United Nations (UN) set 17 sustainable development goals (SDGs) as part of the 2030 Agenda for Sustainable Development (United Nations, 2015). Gender equality is one of the SDGs and is focused on eliminating all forms of discrimination against women. One of the most researched areas of gender equality is the equal integration of women and men into the labor market (Abendroth, 2014). Despite the great progress achieved over the last few years on this issue, women are still paid less than men for similar jobs (Sánchez and Lehnert, 2019). Moreover, women have remained underrepresented in managerial positions and have limited career advancement opportunities (Tonoyan et al., 2020). Surprisingly, studies on gender issues are scant in the extant SE literature (Hossain, 2020).

Ge et al. (2016) found in their study that Uber drivers in Boston gave female passengers longer and more expensive rides. The authors also found that female passengers were constantly exposed to flirting attitudes from Uber drivers. Other cases have been published in news media regarding sexual harassment and assault reported by SE platform users (Levin, 2017; Cramer, 2019). Nevertheless, there is a dearth of research aimed at analyzing gender inequality from the SE’s service providers’ side. Even though gender inequality in the labor market has gained an increasing amount of attention from scholars in management and entrepreneurship areas, it is relevant to explore this research topic in the context of the SE, since SE firms pose different barriers and challenges to service providers that differ from those of traditional firms.

On one hand, SE firms have promoted job flexibility and entrepreneurialism as the main benefits women can attain when working in SEs (Shade, 2018). Certainly, women have found SE an interesting employment alternative to the traditional economy (Singer, 2014). Nevertheless, gender gaps in payment and labor participation are still present in the SE, mainly on ridesharing platforms. A recent study identified that Uber’s male drivers earn 7% more money per hour than female drivers (Cook et al., 2018), and in the United States, only 14% of Uber drivers are women (Huet, 2015). On the other hand, SE transactions occur in intimate settings, which leads to sex and security concerns, especially for women (Schoenbaum, 2016). In this case, women providing services on ridesharing or home-sharing platforms are highly vulnerable to physical and sexual risk, as the interaction between buyer and seller occurs in a private car or home without the presence of other persons (Schoenbaum, 2016).

Based on the aforementioned information, it is timely to deeply comprehend the experiences of women working in SEs. This study is focused on ridesharing services in the context of Mexico, where the economic and social gaps that women face when entering the job market can provide insight into the difficulties of this segment of the population, particularly in developing countries. Mexico reports one of the highest proportions of informal economy activities in Latin America, with only 40% of its employed population working in the formal economy (Gurría, 2019). At the same time, only 45% of Mexican women participate in the job market (Forbes Women, 2021). These numbers are expected to worsen after the COVID-19 pandemic.

## 3 Methodology

The empirical data were collected through in-depth interviews with 25 female drivers with working experience in one of the following ridesharing services: Uber, DiDi, or Cabify (Table 2). These three companies are considered to be the most popular ridesharing apps in Mexico (Bravo Medina, 2019). The number of interviews was determined after saturation of data was reached. Participants were recruited through snowball sampling via personal contacts and respondents’ recommendations. The duration of the interviews ranged between 45 and 60 min.

**Table 2:** List of interviewees.

Code	Age range	City of residency	Period using the ridesharing app
*F1*	25–29	Mexico City	7 months
*F2*	30–34	Mexico City	1 year
*F3*	25–29	Puebla	2 years
*F4*	30–34	Mexico City	1 year
*F5*	30–34	Monterrey	5 years
*F6*	30–34	Monterrey	6 years
*F7*	35–39	Cuernavaca	11 months
*F8*	35–39	Sinaloa	1 year
*F9*	50–54	Puebla	4 years
*F10*	20–24	Monterrey	4 months
*F11*	25–29	Puebla	2 years
*F12*	40–44	Mexico City	2 years
*F13*	30–34	Puebla	2 years
*F14*	25–29	Monterrey	5 years
*F15*	30–34	Monterrey	5 years
*F16*	30–34	Mexico City	3 years
*F17*	45–49	Monterrey	3 years
*F18*	35–39	Monterrey	4 years
*F19*	40–44	Monterrey	4 years
*F20*	25–29	Monterrey	5 years
*F21*	25–29	Monterrey	1 year
*F22*	45–49	Monterrey	6 years
*F23*	30–34	Monterrey	6 years
*F24*	35–39	Mexico City	2 years
*F25*	35–39	Puebla	10 months

A phenomenological approach was adopted to conduct the in-depth interviews. According to Thompson et al. (1989), the main goal of phenomenological interviews is “to attain a first-person description of some specified domain of experience” (p. 138). For phenomenological researchers, the only legitimate source of information is the individual’s account of his/her experiences (Goulding, 2005). In the case of this article, the experiences and views of female drivers working on ridesharing platforms represent the reality of the phenomenon of interest, and hence, the objective is to interpret and find meaning in these experiences. Even though it is recommended to maintain an open and unstructured dialogue with the participant when conducting a phenomenological interview (Thompson et al., 1989), the method of phenomenological interviewing proposed by Bevan (2014) was applied for this study. This method comprises three sections: contextualization, apprehension of the phenomenon, and clarification of the phenomenon. The phenomenological interview began with questions focused on understanding the participant’s context and how she became a driver for a ridesharing app. Then, more descriptive questions were asked to gain an understanding of her work experiences in detail. Last, the imaginative variation technique was applied to unfold the structural components of her experience. The interviews were audio-recorded and transcribed verbatim. A thematic analysis was used to interpret the qualitative data. This inductive method is data-driven, which means that the codification of data is not determined by a pre-existing coding frame, but by the data itself (Braun and Clarke, 2006). After reading the transcripts several times and identifying patterns of meaning within the data, an initial set of codes was created with the aid of MaxQDA. Then the codes were arranged into potential themes. Subsequently, each theme was reviewed and refined by following a two-level process (Braun and Clarke, 2006). First, data extracts for each theme were revised to confirm the internal coherence. Second, the validity of the themes was reviewed in relation to the entire data set. This process went back and forth until generating a rich and final description of the phenomenon of interest (Goulding, 2005). As a form of cross-checking validation, participants were invited to express their opinions regarding the study’s findings.

## 4 Analysis of results

**Figure 1:**
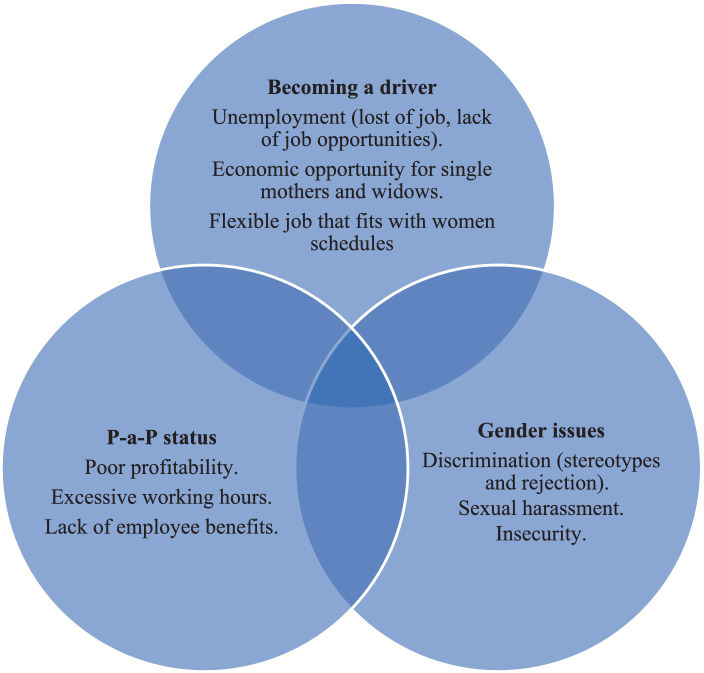
The prosumption experience.

During the analysis of the results, three main aspects were identified (Figure 1) according to the experiences of the interviewees. These aspects span a broad range of motives, barriers and perceived risks female drivers identify within the performance of their job. The first aspect comprises the reasons why women decide to become drivers in a ride-sharing platform (e.g., economic opportunity and flexible schedule). The second aspect, named as “prosumption experience,” addresses the perils of the ambiguous status of their job, since women perceive poor profitability, excessive working hours and a lack of employment benefits for their labor. The third aspect is focused on gender issues female drivers suffer and the ways they cope with these problems.

### 4.1 Becoming a rideshare driver

During the first stage of the phenomenological interview, the participants expressed their reasons and motivations for becoming drivers on a ridesharing platform. Most of them mentioned that unemployment and a lack of job opportunities in the traditional labor market were the main factors that led them to work on these platforms. However, the participants also mentioned that they would not have decided to become drivers for a ridesharing platform if they had a full-time job. In this case, participants do not see their involvement in the SE as a source of extra income or a new business opportunity, but as the only option available to earn money. This is in line with Burtch et al. (2018), who suggest that SE has become a viable job opportunity for unemployed and underemployed individuals. Specifically, women fall into these categories since they represent a vulnerable group in terms of employment access and wage equality (Auspurg et al., 2017).

Another common situation shared by most participants was the need to combine their professional lives with their family care responsibilities. Some participants were single mothers or widows without additional economic support, while others were married women who wanted to contribute to the household economy. A minority of participants were university students with time constraints who were motivated to work on ridesharing platforms to pay their tuition fees. Regardless of their particular experiences, the participants agreed that the possibility of working with a flexible schedule was the main benefit provided by ridesharing platforms. Hence, it is not surprising that SE firms have constantly used the discourse of “labor flexibility” to encourage women to join the SE workforce (Shade, 2018).

*“I needed a job that didn’t force me to be there on set hours because I have a daughter, I am a mother, I am divorced, so I need to be with my daughter and have time for her. So I thought it was the best option because I am my own boss, and I can manage my time and my schedule”—F14*

### 4.2 The prosumer-as-production experience

The second stage of the phenomenological interview enabled a deeper understanding of the participants’ daily experiences as drivers in a ridesharing platform. In a broader sense of the prosumption experience, participants mentioned that they had used the ridesharing app as both passengers and drivers. Based on their experiences, participants could identify more safety concerns from the producer side (driver) than the consumer side (passenger). For instance, before deciding to take a trip, the passenger can see the driver’s information, such as her name, photo, rating, car model, and plate number. When the driver arrives, the passenger can confirm the information with the real identity of the driver. In case of inconsistencies, the passenger can decide to cancel the trip and report the driver to the ridesharing platform.

In contrast, the driver can identify only a few pieces of information about the passenger (e.g., name and rating). After spending time and money to reach the pickup location, the driver can verify the passenger’s information with his/her real identity. However, if the driver finds inconsistencies, she has only two options: to accept the trip to compensate for the time and money she already spent, or cancel the trip and take the risk of being penalized by the ridesharing platform. The latter is more problematic for drivers, since some ridesharing platforms demand a high acceptance rate of rides and consider cancellations to be negative points for drivers’ ratings (Schoenbaum, 2016).

*“I think that there is a difference because you as a customer know more about who is driving you, you see the picture, plate numbers, which car is it, you can keep a registry, you can say ‘this person is taking me there’, right? But whenever you are behind the wheel, you don’t know anything. They can tell you ‘You’re picking up Maria’, it’s what they usually say, or ‘You’re picking up Maria at this address’ and you see if you accept the trip, but you arrive for Maria and it turns out that she ordered the trip for her cousin or friend and a man gets on the car. Then, you don’t know anything about that person, you are much more exposed”—F3*

As mentioned at the beginning of this section, the main motives for female participants to become drivers in a ridesharing platform were the need for income and a flexible and autonomous work schedule. However, their working experiences depicted a different reality more related to the exploitative nature of prosumer capitalism in the SE described by Ritzer (2015). First, participants noted that ridesharing platforms’ fees have risen in recent years. In Uber’s case, drivers must pay 35% of their earnings to the platform, while in other platforms (Didi and Cabify), the fee rates are between 15% and 20%. Coupled with this, participants complained about the constant decline of ride prices due to the platforms’ price-cutting campaigns in order to attract new customers and the increase in the number of new drivers signing up to the platforms. Under these circumstances, participants acknowledged that working on a ridesharing platform is not as profitable as it was in the initial years.

A particular situation faced by drivers on a ridesharing platform is the use of their own resources to perform the job. Participants mentioned a myriad of expenses that affected their long-term income. For instance, the driver must buy a brand-new car according to the ridesharing platform’s requirements. The driver also absorbs the costs associated with car maintenance, insurance, registration fees, and gasoline. In addition, hidden costs, such as cellphones, car depreciation, and repairs in the case of accidents, are not covered by the platform. As Bardhi and Eckhardt (2017) contend, prosumption in the SE is exploitive since the service provider is not compensated by his/her labor. The authors also argue that service providers in the SE are forced to be entrepreneurial, which means that they must use their own resources to make ends meet, and thus, it can affect their relationship with their possessions.

*“There was a lot of competition and now with the pandemic, the prices went down and so did our profit. It was a lot less, I mean, maybe they charge the customer the same, but they take a lot in taxes from us. (…) Per ride, Uber takes around 35%, plus the taxes we pay, then the gas, then there’s almost nothing left. (…) Everything related to the car, the tires, everything, absolutely everything, it’s on us as the driver, the maintenance, if anything breaks down, it is on us the drivers. (…) Right now we keep maybe 25% or 30%, if that.”—F15*

Another exploitive dynamic of the SE that was evident in the participants’ statements was the work schedule. Even though participants said that their initial involvement with ridesharing platforms was motivated by the option of working on a flexible schedule, they admitted working between 10 and 15 h per day to make a profit. Indeed, the algorithm seems to dictate the pace and intensity of working for the drivers. Participants explained that the algorithm makes it difficult to calculate the cost–benefit balance of accepting a ride, as the ride’s distance is not the main indicator to determine its price. For instance, a short and long ride can have a similar price for the passenger depending on the algorithm, although a longer ride would be more expensive for the driver. Chai and Scully (2019) adopted the term “invisibility of owners” to explain how the algorithm facilitates the extraction of the surplus from service providers’ labor by SE platform owners. Given that service providers know little about how the algorithm sets ride prices, evaluates drivers’ performance, and distributes economic gains, their capacity for contestation and retaliation against this process is limited (Chai and Scully, 2019).

In summary, the flexible working schedule is mainly controlled by the demand and the algorithm and, to a lesser extent, by the service providers (Jin et al., 2018). Participants expressed that to make more money from their labor, they needed to work in times of high demand, commonly during nights and weekends. However, they felt more at risk, especially at night, as they could be an easy target for sexual assaults and violence. Hence, female drivers face a difficult situation in which they are forced to work long hours in a context with a high level of uncertainty due to both the dynamic pricing mechanism of the ridesharing platform and the risks of the working context.

*“When I started, like 2 years ago, it was around 12 or 15 hours so it was suitable (…) Monday and Friday (I would drive) 8 hours because those are calmer days, but Wednesday to Sunday are the best days for us”—F2*

Regarding the drivers’ status, ridesharing platforms treat drivers as independent contractors, rather than employees. This status negatively affects drivers, as they do not receive benefits common for full-time workers, such as paid vacations and health and retirement plans (Ganapati and Reddick, 2018). In the participants’ remarks, it was evident to identify diverse problems and dilemmas related to the lack of support and legal responsibility from ridesharing platforms. For instance, the participants mentioned that ridesharing platforms provide minimal assistance in case of accidents, which is a latent risk when performing the job. Indeed, one of the greatest fears of participants was to be assaulted and a victim of car theft. Overall, participants recognized a high level of uncertainty when working on a ridesharing platform, as they are fully responsible for most of the problems associated with their job.

*“You know the platform doesn’t give you any benefits (…) You don’t have anything, you don’t make a career in the platform, no social security, payroll receipts, nor anything. And you see, whenever you want to ask for credit or something, the first thing they ask for is payroll receipts.”—F20*

Participants also mentioned that their jobs involved risks derived from the context of violence in Mexico. Most of the participants agreed that ridesharing platforms do not provide adequate mechanisms to protect and safeguard their integrity. For instance, in Mexico, ridesharing platforms enable paying with cash, increasing the probability of drivers being assaulted. Another complaint expressed by the participants was the lack of training programs and support by ridesharing platforms in terms of avoiding dangerous areas and what to do in case of assault. Indeed, some participants shared their experiences of being assaulted because of their unawareness about driving in areas with high crime rates. These participants also revealed that the ridesharing platform did not cover medical costs related to the physical injuries suffered during the assault.

*“I was robbed a month ago and they beat me up, so that’s why I decided to work less through platforms and do only airport services. (…) Didi doesn’t give you any support. They robbed me of around 1000 pesos (50 USD), and Didi doesn’t give you anything back, even though it was one of their users. (…) That’s why the situation is so dangerous, they rob you or something and you don’t have any insurance, you know? I mean, ‘hey, someone robbed you, they took this much, I’ll make up for it’, maybe not even the full amount, but something that helps you feel supported. However, Didi only sends you an email saying ‘We’re sorry that happened to you’, and that’s it”—F21*

### 4.3 Gender discrimination and sexual harassment in the SE

One of the most interesting and simultaneously shocking topics of the phenomenological interviews was the specific risks and problems faced by female drivers. In addition to the problems associated with wage instability, long working hours and lack of job benefits, participants also described multiple acts of gender discrimination and sexual harassment from passengers. Gender discrimination, defined by Pietiläinen et al. (2020) as an “unfair treatment caused by prejudices related to gender” (p. 311), was reflected in the participants’ experiences in different manners, such as passengers canceling rides without a reason and passengers making discriminatory comments during the ride about the poor capacity of the female driver to perform her job because of her gender. It is worth mentioning that these gender discriminatory acts were perpetrated by both men and women, which means that gender discrimination in this type of work is strongly rooted in Mexican culture.

*“A lot of users put it like ‘and you’re not scared?’ or ‘for being a woman you drive alright’ or even ‘sorry but when I saw you were a woman I thought of cancelling, but it was too late so I had to take the ride with you’—F11*

Participants also shared shocking stories about diverse acts of sexual harassment they suffered from passengers. The stories are varied, including verbal threats of sexual violence, unwelcome sexual advances (e.g., one passenger kissed the driver without her consent), and requests for sexual favors (e.g., a couple proposed a threesome to the driver). The prevalence of violence against women in Mexico exposes female drivers to multiple risks, and unfortunately, ridesharing platforms have not yet provided effective measures to protect them. Some ridesharing platforms have implemented an exclusive pickup service for women, but only passengers can choose the gender of the driver and not vice versa. Therefore, some participants said that they opted not to work during the night to avoid these risks. However, their capacity to profit is curtailed by this strategy, since the algorithm commonly sets higher prices for night rides.

*“They are disgusting, utterly disgusting. A user didn’t want to get out of the car and kept telling me that if I were to go home with him he would give me 60 pesos (3 USD)”—F18*

*“A guy harassed me. (…) We arrived at the destination and you expect them to get out, but he sat in the middle seat in the back, pulls me, and kissed me. That was one. I reported it and they said they would review it, but nothing happened, he wasn’t even suspended. Another time, a couple that was leaving a club (…) began to tell me to take them to my place, they wanted me to participate in a threesome with them. I was very uncomfortable, and I asked them to get out of the car; otherwise, I would call the police. I reported it to Uber, but they suspended both accounts (driver’s and customer’s account), supposedly while they were investigating. They left me without a job for a week, without access to my account. In addition to being harassed, Uber oppressed me by taking my access to my account and leaving me without a job. (…) If you want to denounce what happened you need to have at least the customer’s name, and they didn’t even give me the name”—F1*

## 5 Theoretical discussion

Based on the analysis of the narratives and the experiences provided by the participants, it was possible to identify four main topics of interest: the decision to become a driver for a ridesharing platform, the p-a-p experience, gender issues at work (gender discrimination and sexual harassment), and coping strategies to deal with the exploitative SE system. In turn, these topics were helpful to gain insights into p-a-p behavior on ridesharing platforms from a gender perspective. Regarding the first topic, women perceive ridesharing platforms as an easy way to make money out of the traditional labor market because of the flexible work schedule and the low entry barriers to start working. However, these benefits do not go unnoticed by ridesharing platforms, which have embraced them as core elements in their promotional activities and discourses targeted to women.

Shade (2018) contended that the strategy followed by SE platforms of communicating “the empowering benefits of labor flexibility and entrepreneurialism for women” (p. 37) reinforces the ideology of neoliberal feminism. In contrast with the liberal feminism movement that exhorted social solidarity against and awareness of the gender inequalities of the system, neoliberal feminism places full responsibility on the individual woman who must take care of her own well-being and professional development (Rottenberg, 2014). This empowering rhetoric hides a darker reality, where women’s needs are not a priority for SE platforms. For instance, Uber has been the focus of criticism due to its unsafe and precarious labor conditions for women (Shade, 2018). A closer look at these promotional activities deployed by different SE platforms is needed to identify the different discourses they use to attract women to their workforce.

The second topic yields interesting insights into the p-a-p experiences of female drivers on ridesharing platforms. First, it seems that most of the participants’ concerns about their working conditions, including volatile fares and long working hours to make profits, were derived from an algorithmic control exerted by ridesharing platforms. Indeed, algorithmic management can be considered a form of digital control that places the customer at the core of all the platform’s activities. Hence, service providers’ behaviors are structured by customers’ needs, rather than the manager’s orders (Wood et al., 2019). Uber is a clear example of this type of “soft control” over the workforce through algorithmic management (Wood et al., 2019). In a similar vein, Gerwe and Silva (2020) noted that “the negative aspects of reliance on [the] algorithm seem to be more pronounced in the case of labor platforms, especially Uber” (p. 90–91).

Therefore, in the context of ridesharing platforms, the algorithm makes the hands of the owners of the capital less visible, who can easily extract the surplus of service providers’ labor without facing any resistance from their part (Chai and Scully, 2019). A similar idea was explored by Ritzer (2015), who argued that prosumer capitalism and its double exploitative system were expanding to different arenas, including the SE. Ritzer (2015) warned that capitalists are always finding new ways to exploit prosumers, who do not perceive that they are exploited and alienated by the system due to their lack of knowledge about this process. Participants’ remarks clearly reflect this phenomenon, as they feel that the platform prioritizes the security and needs of customers over theirs, and that the functioning of the algorithm is difficult to understand and predict. Even though they hold a shared perception about these problems, they do not have a full understanding of their disadvantageous position in the system.

Second, the use of participants’ own resources to perform their jobs on ridesharing platforms exposes another exploitative mechanism of SEs, in which service providers are forced to be entrepreneurial while holding insecure jobs (Bardhi and Eckhardt, 2017). This entrepreneurial spirit was clearly identified by Peticca-Harris et al.’s (2020) study on Uber drivers, who justified their decision to work for Uber by using their own resources as a way to build a promising future in the present. However, the authors concluded that Uber is not an alternative to capitalism, but an exploitative version of it that takes advantage of a precarious workforce and regulatory ambiguity to succeed. In this case, the concept of the prosumer is crucial to understanding the different dynamics and effects concerning the position of service providers in the SE. Bardhi and Eckhardt (2017) contend that the SE can potentially negatively affect the consumption identity and orientations of p-a-p’s. The current study also sheds light on the multiple resources p-a-p’s require to invest and use on their own to perform their jobs without the financial support of the ridesharing platform.

Finally, the third topic is centered on gender issues, which directly affect female drivers working on ridesharing platforms. In addition to the problems related to the p-a-p status, female drivers also face specific problems, such as sex-based violence, gender discrimination, and sexual harassment, while performing their job. First, the context of the study is Mexico, the country with the highest rate of femicides in Latin America, along with Brazil (CEPAL, 2019). At the same time, Latin America has been regarded as one of the most important and promising markets for ridesharing platforms due to its large population concentrated in large cities (e.g., Mexico City and São Paulo) with insufficient public transportation systems (Lustig, 2018). Under these circumstances, participants found it convenient, yet unsafe, to work on ridesharing platforms. Participants expressed that while learning how and where to drive in the city to avoid dangerous areas, they had bad experiences related to assaults and physical aggression. In these cases, ridesharing platforms offered minimal assistance regarding training and immediate support for accidents.

Second, gender discrimination is another problem faced by female drivers. Schoenbaum (2016) argues that the intimate nature of SE transactions leads to gender discrimination. For instance, a service provider’s identity and physical characteristics are significant pieces of information customers use to evaluate the entire service process, which in turn exacerbates the generation of stereotypes. For female drivers, the stereotype of car driving as a masculine practice has negative implications on their job performance. Participants mentioned that they had diverse experiences related to gender discrimination, such as sudden cancelation of rides and gender-based comments made by passengers related to their limited driving skills. It is worth mentioning that ridesharing platforms have attempted to implement strategies aimed at preventing gender discrimination, but it is crucial to expand and reinforce these efforts toward female drivers and passengers alike.

Third, sexual harassment has become a major issue in ridesharing services. A report by Uber revealed that 3,045 sexual assaults (ranging from nonsexual aggressions to rapes) occurred during rides in the United States in 2018, and more cases with different ridesharing platforms have been widely reported in China, India, and Brazil (Conger, 2019). Female drivers are also exposed to sexual harassment from passengers, but the most concerning issue with ridesharing platforms is that the same system perpetuates this situation. Schoenbaum (2016) contends that “a combination of market forces and firm policies mean that female drivers suffer financially when they try to mitigate safety concerns” (p. 26). In this case, if a female driver rejects the majority of male passengers (particularly during nighttime) to avoid risks, then her financial gains will decline significantly. In addition, ride cancellations are counterproductive for female drivers, since bonuses require acceptance rates higher than 90%, and ride cancellations can negatively affect drivers’ ratings (Schoenbaum, 2016).

## 6 Practical implications

Ridesharing platforms can take the lead to improve the conditions of women drivers by implementing specific lines of action. First, women drivers in ridesharing platforms are forcibly drawn to keep a low rejection rate for passengers as the opposite could negatively affect their ratings and income overall. A more flexible rejection rate could improve the conditions of women drivers in terms of safety and autonomy. Moreover, ridesharing platforms may give an extra financial incentive to women drivers each time they pick up women passengers. Both mechanisms can work together to empower women drivers without affecting their profits.

Second, ridesharing platforms should develop training programs and workshops for women aimed at improving their skills and abilities as drivers. Most of the women who decide to start working as drivers in ridesharing platforms have no prior experience in this job activity. It is important to point out that men and women alike are prone to car accidents. Indeed, it has been proved that men drivers are more likely to engage in riskier behaviors while driving than women drivers (Insurance Institute for Highway Safety, 2021). Regardless the gender difference, a recommended course of action is to develop a set of training programs aimed at reducing potential driving threats as well as enhancing the experience of women drivers.

Third, ridesharing platforms must focus their efforts on properly attending and following-up women drivers’ problems related with gender discrimination and sexual harassment. During the interviews, participants mentioned that they did not feel supported by ridesharing platforms when reporting an act of discrimination or sexual harassment. Ridesharing platforms’ commitment to the protection and empowerment of women drivers should begin with a strong anti-gender-based violence policy. In addition, online seminars and about diverse gender issues can aid women drivers to effectively deal with these situations.

Lastly, ridesharing platforms should also engage in educational efforts targeted to the audience with the main aim of creating awareness regarding gender issues of women drivers. As clearly identified in the analysis of results, there are implicit discriminatory acts that most of the time are not fully perceived or consciously understood by the passengers who perpetuate them. Hence, ridesharing platforms can generate advertising campaigns with a social marketing focus, that is, with the intention of changing negative beliefs, attitudes and behaviors of passengers regarding women drivers skills and capacity.

## 7 Conclusion

This paper explored the paradox of the SE and the p-a-p phenomenon from the perspective of female drivers in ridesharing services. This analysis provides insights for understanding the experiences of drivers from a gender perspective by drawing on Ritzer’s (2015) theory of prosumer capitalism. Using this analysis, four main areas were identified: the process before the decision of becoming a driver, usually guided by the need for flexible employment; the P-a-P experience of dealing with unsafe work conditions, algorithm management, and user experience prioritization; gender issues at work, such as discrimination, harassment, and violence; and the coping strategies the drivers develop to deal with the exploitative SE system, which relies mostly on gender-segregated networking.

Notwithstanding, the current study is not exempted from limitations that can lead to potential lines for future research. While this research provides a phenomenological approach to the topic of interest, quantitative studies could add additional insights into the experience of service providers in SEs. Another area for further research is the cultural and institutional differences that may arise from different contexts; more specifically, a comparative study of developing and developed countries from different regions would provide a deeper understanding of the possible variants that affect women drivers in ridesharing services. Moreover, further research could explore the p-a-p experience of women in different SE sectors, such as the ever-growing food delivery services. Lastly, future studies may delve into the evolving landscape of local laws and regulations concerning service providers within the sharing economy. Research opportunities may focus on liabilities, rights and regulatory challenges to ensure equitable treatment and safeguard worker interests.

## Data availability statement

The datasets presented in this article are not readily available because information is confidential. Requests to access the datasets should be directed to frcastillo@up.edu.mx.

## Ethics statement

Ethical approval was not required for the study involving human participants as the Divisions of Social Sciences and Management at the Universidad Anáhuac Mayab do not have an institutional ethics committee. The Department of Research and Development confirmed that the study was conducted in accordance with the local legislation and institutional requirements. The participants provided written informed consent for participation in the study and for the publication of any potentially/indirectly identifying data included in the article.

## Author contributions

FC-V: Conceptualization, Formal analysis, Methodology, Writing – original draft. RC-V: Data curation, Writing – review & editing. KC-V: Funding acquisition, Resources, Writing – review & editing.
